# Caloric vestibular stimulation for the management of motor and non-motor symptoms in parkinson's disease: Intention-to-treat data

**DOI:** 10.1016/j.dib.2019.104228

**Published:** 2019-07-08

**Authors:** David Wilkinson, Aleksandra Podlewska, Sarah E. Banducci, Tracy Pellat-Higgins, Martin Slade, Mayur Bodani, Mohamed Sakel, Lanty Smith, Peter LeWitt, Kristen Ade

**Affiliations:** a School of Psychology, University of Kent, Canterbury, UK; b Scion NeuroStim, LLC, Durham, NC, USA; c Centre for Health Services Studies, University of Kent, Canterbury, UK; d Yale University, School of Public Health, New Haven, CT 06510, USA; e Neuropsychiatry Service, Kent & Medway NHS and Social Care Partnership Trust, UK; f East Kent Neuro-Rehabilitation Service, East Kent Hospitals University NHS Foundation Trust, Canterbury, UK; g Parkinson's Disease & Movement Disorders Program, Henry Ford Hospital, Wayne State University School of Medicine, West Bloomfield, MI 48322, USA

**Keywords:** Non-invasive brain stimulation, Sensory neuro-modulation, Neuro-rehabilitation, Cognition

## Abstract

This report provides data related to the safety and effectiveness of repeated time-varying caloric vestibular stimulation (CVS) as a treatment for motor and non-motor features of Parkinson's disease (PD). Forty-six subjects receiving stable anti-Parkinsonian therapy were randomized to active (n = 23) or placebo (n = 23) treatment arms. Subjects self-administered CVS twice-daily over a period of 8 weeks at home via a portable, pre-programmed, solid-state ThermoNeuroModulation (TNM™) device delivering continually-varying thermal waveforms through aluminium ear-probes mounted on a wearable headset. Change scores from baseline to end of treatment and to a 1-month follow-up were determined using standardized clinical measures. The data presented here report sample demographics, detailed safety data and the statistical outcomes from the intention-to-treat and modified intention-to-treat analyses. **These data supplement findings of the main per protocol analysis reported in the allied article entitled, ‘Caloric Vestibular Stimulation for the Management of Motor and Non-Motor Symptoms in Parkinson's Disease’ Wilkinson et al**.

**Table:** Specifications table

Subject area	*Clinical Neurology*
More specific subject area	*Rehabilitation*
Type of data	*Tables and figures.*
How data was acquired	*Data were acquired using standardized clinical measures including:* *The Movement Disorder Society - Unified Parkinson's Disease Rating Scale, The Non-Motor Symptom Scale, The Montreal Cognitive Assessment Scale, The Hospital Anxiety and Depression Scale, The Epworth Sleepiness Scale, The Fatigue Severity Scale, The Timed-up-and-go test, The 10-m walk test, the 2-min walk test, The Parkinson's Disease Questionnaire – 39, The Modified Schwab and England Activities of Daily Living Scale,* *The EQ-5D-5L and the SF-12 Health Survey. Safety data were acquired by means of subject-provided adverse event reports.*
Data format	*Analyzed*
Experimental factors	*Subjects were randomized 1:1 to an active or placebo treatment arm.*
Experimental features	*Clinical assessments were repeatedly administered by a blinded assessor during a 4-week pre-treatment baseline, 8-week treatment period and a 5-week post-treatment period. Subjects self-administered twice daily treatments using a solid-state device.*
Data source location	*Clinical assessments were performed in subjects' homes within southeast England, UK.*
Data accessibility	https://data.mendeley.com/datasets/m7ths6gdv9/1
Related research article	*This article accompanies:* Wilkinson, D., Podlewska, A., Banducci, S., Pellat-Higgins, T., Bodani, M., Sakel, M., Smith, L., LeWitt, P., Ade K., Caloric Vestibular Stimulation for the Management of Motor and Non-Motor Symptoms in Parkinson's Disease. *Parkinsonism & Related Disorders*, in press. https://doi.org/10.1016/j.parkreldis.2019.05.031

**Table:** 

**Value of the data** • The intention-to-treat analyses help clinicians understand the effectiveness of the treatment. • The sample demographics help clinicians understand who may benefit from the treatment. • The adverse event data help clinicians understand treatment tolerability and how it contrasts with the more commonly applied method of caloric vestibular stimulation which involves chilled water irrigation and is used to diagnose balance disorders and brainstem dysfunction.

## 1 Data

**Fig. 1:**
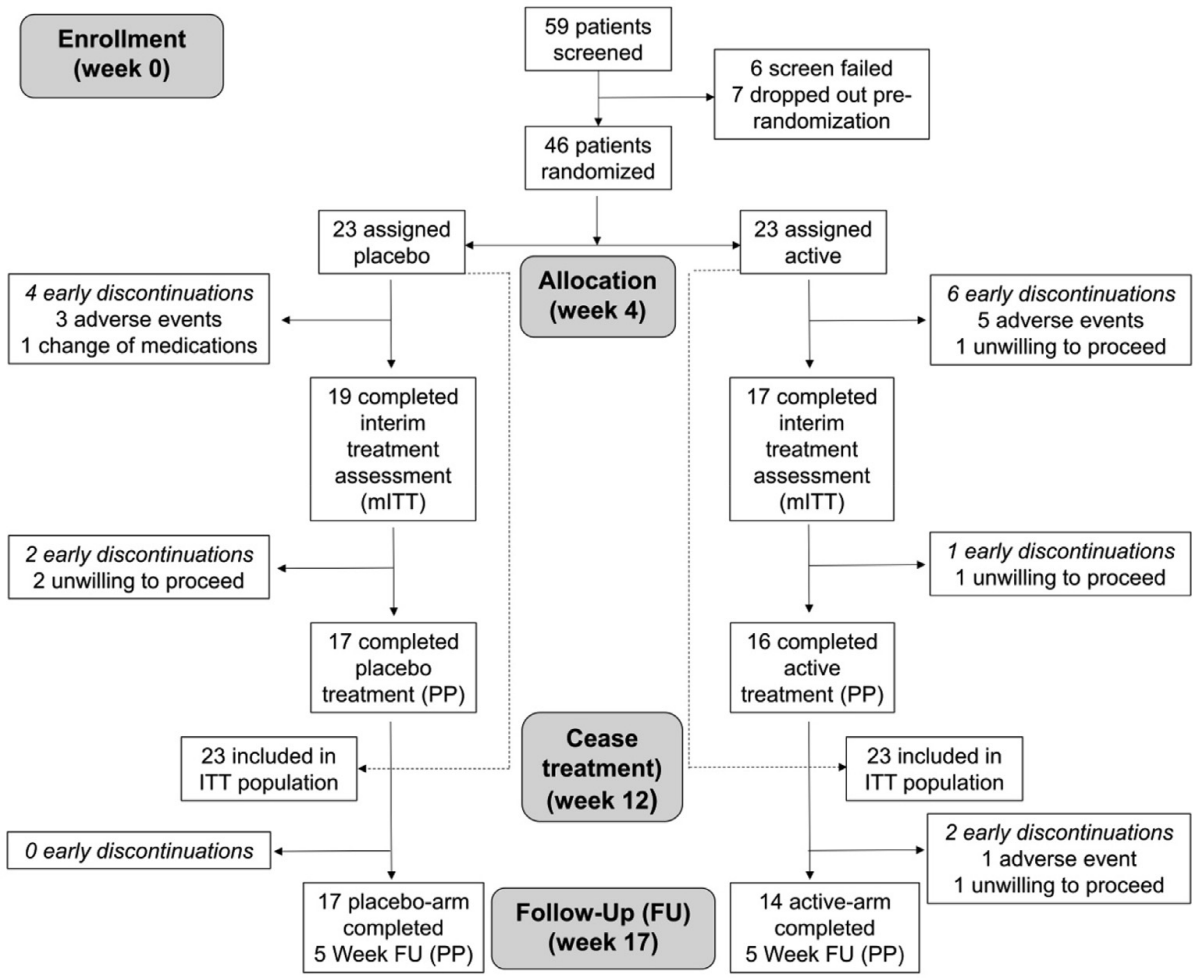
Consort flow diagram.

**Fig. 2:**
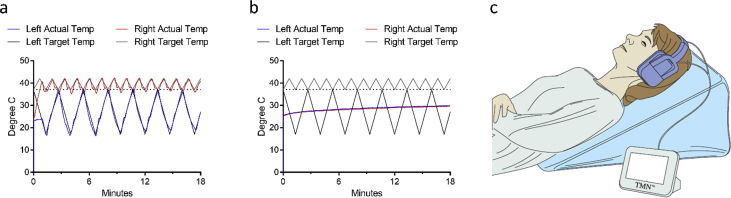
Target and actual thermal waveform profiles recorded by thermistors located within the aluminium ear pieces for **a)** an active treatment and **b)** a placebo treatment, and **c)** a schematic of a patient undergoing treatment while wearing the CVS headset and lying on an incline wedge pillow. Schematic: Copyright (2016) Wiley. Used with permission from [1].

These data describe the demographic profile of all randomized subjects as well as measures evaluating the safety and effectiveness of the intervention. Fig. 1 shows the number of participants randomized and subsequently entered into the per-protocol, intention-to-treat, and modified intention-to-treat (i.e. participants who completed the first but not second scheduled assessment during treatment) analyses. Fig. 2 shows the thermal profiles of the stimulation waveforms received by active and sham participants and also illustrates an individual undergoing treatment. Table 1 provides the demographic and clinical characteristics of the participants at baseline. Table 2, Table 3 show the intention-to-treat and modified-intention-to-treat statistical outcomes. Table 4 summarizes the adverse events and their likely relation to study participation.

**Table 1:** Study demographics and baseline measures. + indicates non-normal distribution of data; in these cases, the reported values are (median ± range low value, high value). Baseline scores provide the average of the two baseline measures. The VAS provides subjects' perceived effectiveness of their current dopamine replacement therapy (DRT) (1–10).

	Intention-To-Treat			Modified Intention-To-Treat		
	Active	Placebo	p-value	Active	Placebo	p-value
	23	23		17	19	
**Demographics (mean (SD))**						
Age (y)	69.7 (11.3)	72.2 (6.6)	0.361	68.2 (11.6)	72.1 (7.1)	0.230
Male sex, No. (%)	12 (52.2%)	18 (78.3%)	0.063	11 (64.7%)	16 (84.2%)	0.177
Years of education+	14.0 (12.0, 21.0)	14.0 (12.0, 22.0)	0.676	14.0 (12.0, 21.0)	14.0 (12.0, 22.0)	0.830
Time since PD diagnosis (y)+	11 (2, 28)	5 (2, 14)	0.003	9 (2, 28)	5 (2, 14)	0.020
Time on DRT (y)+	11 (1, 28)	5 (2, 14)	0.004	9 (1, 28)	5 (2, 14)	0.027
VAS score+	7 (1, 10)	6 (1, 10)	0.456	7 (1, 10)	6 (1, 10)	0.409
**Baseline assessment scores (average ± SD)**						
MDS-UPDRS total score						
Part I	17.2 (5.7)	15.1 (4.9)	0.190	16.9 (5.8)	16.0 (4.9)	0.615
Part II	22.4 (9.1)	21.4 (5.3)	0.637	22.1 (9.8)	21.6 (5.7)	0.870
Part III	49.3 (17.6)	45.4 (16.3)	0.433	47.6 (16.9)	44.1 (16.3)	0.530
Hoehn Yahr +	2.5 (1.5, 4.0)	2.0 (1.5, 4.0)	0.393	3.0 (2.0, 4.0)	2.0 (2.0, 4.0)	0.298
PIGD	8.1 (3.9)	7.5 (4.3)	0.604	8.1 (4.5)	7.2 (4.6)	0.564
NMSS total score	126.2 (29.8)	117.7 (32.6)	0.364	127.4 (31.1)	125.1 (30.8)	0.825
Domain 1: Cardiovascular/falls *	0.0 (0.0, 12.0)	2.0 (0.0, 10.5)	0.313	0.0 (0.0, 12.0)	0.5 (0.0, 10.5)	0.514
Domain 2: Sleep/fatigue	23.2 (7.0)	19.3 (8.5)	0.094	22.2 (6.1)	20.2 (8.9)	0.464
Domain 3: Mood/cognition	23.1 (12.1)	25.5 (16.1)	0.576	23.1 (12.4)	26.9 (16.4)	0.449
Domain 4: Perceptual/hallucinations+	0.0 (0.0, 16.0)	0.0 (0.0, 19.5)	0.774	0.0 (0.0, 16.0)	0.0 (0.0, 15.0)	0.509
Domain 5: Attention/memory+	13.0 (1.0, 36.0)	13.0 (1.0, 34.5)	0.983	15.3 (11.5)	14.9 (9.3)	0.922
Domain 6: Gastrointestinal tract	12.5 (8.2)	11.0 (6.6)	0.498	12.4 (8.8)	12.1 (6.3)	0.914
Domain 7: Urinary	18.4 (9.0)	15.3 (8.8)	0.242	20.7 (8.1)	16.5 (9.0)	0.161
Domain 8: Sexual function+	19.5 (1.0, 24.0)	18.0 (1.0, 24.0)	0.375	21.0 (1.0, 24.0)	19.0 (1.0, 24.0)	0.523
Domain 9: Miscellaneous	12.5 (6.1)	11.6 (6.7)	0.640	11.7 (6.3)	13.0 (6.2)	0.545
MoCA+	25.0 (18.5, 28.5)	24.5 (10.0, 29.0)	0.415	25.0 (20.5, 28.5)	24.5 (10.0, 29.0)	0.465
HADS Anxiety+	4.0 (1.0, 14.5)	4.5 (1.0, 13.0)	0.692	4.0 (1.0, 14.5)	5.0 (1.0, 13.0)	0.365
HADS Depression+	6.5 (2.0, 14.5)	6.5 (2.5, 16.5)	0.904	6.5 (2.0, 14.5)	6.5 (2.5, 16.5)	0.535
Epworth Sleepiness Scale	13.1 (5.5)	10.8 (5.9)	0.170	14.4 (5.4)	10.1 (6.0)	0.032
Fatigue Severity Scale	48.9 (6.7)	39.9 (11.3)	0.002	47.8 (6.6)	41.1 (10.7)	0.034
TUG +	14.1 (7.6, 59.1)	11.4 (6.5, 213.0)	0.983	14.1 (7.6, 27.1)	11.4 (6.5, 213.0)	0.950
10 m walk self-paced+	9.2 (5.0, 29.8)	7.2 (4.3, 38.7)	0.330	9.3 (5.3, 29.8)	6.6 (4.3, 38.7)	0.204
10 m walk fast-paced	5.5 (1.4)	4.7 (1.2)	0.083	5.2 (1.1)	4.6 (1.2)	0.213
2 minute walk	73.2 (25.3)	77.4 (33.1)	0.642	73.9 (27.0)	82.8 (32.7)	0.393
PDQ-39	34.8 (12.2)	30.8 (8.8)	0.200	32.4 (11.3)	30.3 (8.3)	0.528
Modified Schwab & England+	70.0 (32.5, 85.0)	65.0 (40.0.95.0)	0.434	70 .0 (32.5, 85.0)	65.0 (40.0, 95.0)	0.656
EQ-5D-5L	0.63 (0.12)	0.66 (0.11)	0.325	0.64 (0.13)	0.65 (0.08)	0.696
SF-12 PCS	31.8 (7.2)	36.3 (5.4)	0.020	32.6 (6.2)	35.8 (5.4)	0.102
SF-12 MCS	48.4 (9.7)	45.1 (9.0)	0.233	49.1 (10.5)	45.8 (8.5)	0.308

**Table 2:** Intention-to-treat statistical results. Therapeutic gains are calculated as Active - Placebo group change scores. +Medians and median difference (based on all possible differences) and non-parametric Hodges-Lehmann confidence intervals. *Statistically significant (p < 0.05), § exceeds minimal clinically important difference (MCID). End of treatment assessment (week 12), 5 week post-treatment follow up assessment (week 17). Abbreviations: Non-Motor Aspects of Experiences of Daily Living (nM-EDL), Motor Aspects of Experiences of Daily Living (M-EDL). □ Denotes normal distributions at week 12 and non-normal distributions at week 17. ◊ Denotes non-normal distributions at week 12 and normal distributions at week 17.

Outcome Measure	Change from baseline at week 12				Change from baseline at week 17			
	Active Mean	Placebo Mean	Therapeutic Gains	95% CI	Active Mean	Placebo Mean	Therapeutic Gains	95% CI
*Sample size*	*23*	*23*			*23*	*23*		
Movement Disorder Society Sponsored Unified Parkinson's Disease Rating Scale								
Part I: nM-EDL+	−3.0	−1.0	−2.0	−5.5 to 0.0	−3.9	−0.4	−3.5*§	−5.9 to −1.1
Part II: M-EDL	−3.4	−0.5	−2.9*	−4.9 to −0.9	−3	−0.2	−2.7*	−5.3 to −0.7
Part III: Motor exam	−8.5	−3.1	−5.3*§	−10.6 to 0.0	−9.2	1.7	−10.9*§	−16.4 to −5.5
Postural Instability/gait difficulty +	0.0	0.0	−0.5	−1.5 to 0.5	−0.5	0.0	−1.0*	−2.0 to 0.0
Non-Motor Symptom Scale								
Total Score+	−33.5	0.0	−28.5*	−43.5 to −6.5	−37.5	1.0	−32.5*	47.0 to −13.0
Domain 1: Cardiovascular +	0.0	0.0	0.0	0.0 to 3.0	0.0	0.0	0.0	0.0 to 1.5
Domain 2: Sleep/fatigue+	−2.5	−1.0	−2.0	−5.0 to 2.0	−5.0	0.0	−2.5*	−7.0 to 0.0
Domain 3: Mood/cognition	−7.2	2.1	−9.3*	−15.5 to −3.2	−6.9	2.8	−9.7*	−15.6 to −3.7
Domain 4: Perceptual +	0.0	0.0	0.0	0.0 to 0.0	0.0	0.0	0.0	0.0 to 0.0
Domain 5: Attention/memory□	−3.9	0.4	−4.3*	−8.0 to −0.6	−1.0	0.0	−4.5*	−11.5 to 0.0
Domain 6: Gastrointestinal tract◊	−2.5	0.0	−2.0	−5.5 to 1.0	−3.4	−0.9	−2.6*	−5.5 to 0.4
Domain 7: Urinary□	−4.7	−0.5	−4.2*	−7.9 to −0.4	−3.0	0.0	−3.0	−7.5 to 0.0
Domain 8: Sexual function +	0.0	0.0	−2.5*	−3.0 to 0.0	0.0	0.0	−3.0*	−4.0 to 0.0
Domain 9: Miscellaneous	0.0	0.0	0.0	−2.0 to 1.5	0.0	0.0	−1.0	−3.5 to 1.0
Montreal Cognitive Assessment								
Total Score +	1.5	0.0	1.5*	0.0 to 2.5	1.5	0.0	1.5*	0.5 to 3.0
Hospital Anxiety and Depression Scale								
Anxiety Score+	0.0	−0.5	0.0	−2.0 to 0.5	0.0	0.0	−1.0	−2.5 to 0.0
Depression Score+	−0.5	0.0	−0.5	−1.5 to 0.0	0.0	0.0	−1.0	−3.0 to −0.0
Epworth Sleepiness Scale								
Total Score□	−1.5	0.2	−1.7	−3.7 to 0.3	0.0	0.0	−0.5	−2.5 to 1.0
Fatigue Severity Scale								
Total Score	−3.4	−1.1	−2.4	−8.0 to 3.2	−3.4	−1.1	−2.4	−8.0 to 3.2
Timed-up-and-go								
Time to complete +	−1.1	0.0	−1.3	−3.7 to 0.5	−0.9	0.0	−0.7	−2.7 to 1.1
10 m walk								
Self-paced walk time (seconds) +	−0.6	0.0	−0.6*	−1.2 to −0.7	−0.6	0.1	−1.0*	−1.7 to −0.4
Fast-paced walk time (seconds)	0.2	0.2	0.0	−0.4 to 0.4	0.2	0.3	−0.0*	−0.4 to 0.4
2 minute walk								
Distance (meters)	0.3	−1.7	2.0	−7.7 to 11.8	4.8	−3.8	8.6	−0.4 to 17.5
Parkinson's Disease Questionnaire - 39								
Summary Index Score+	−6.6	−0.4	−2.6	−7.0 to 0.6	−6.9	−2.3	−4.6	−9.3 to 0.0
Modified Schwab and England Activities of Daily Living Scale								
Total Score+	4.0	0.0	4.0	0.0 to 10.0	5.0	0.0	5.0*	0.5 to 10.0
EQ-5D-5L								
Index Score◊	0.02	0.01	0.02	−0.03 to 0.08	0.01	−0.01	0.03	−0.05 to 0.10
SF-12 Health Survey								
Physical Composite Score◊	1.7	0.0	2.1	−0.9 to 6.4	2.1	0.6	1.6	−2.5 to 5.7
Mental Health Composite Score◊	0.0	0.0	1.8	−2.4 to 5.5	2.2	−0.9	3.0	−1.4 to 7.4

**Table 3:** Modified intention-to-treat statistical results. Therapeutic gains are calculated as Active - Placebo group change scores. +Medians and median difference (based on all possible differences) and non-parametric Hodges-Lehmann confidence intervals. *Statistically significant (p < 0.05), § exceeds minimal clinically important difference (MCID). End of treatment assessment (week 12), 5 week post-treatment follow up assessment (week 17). Abbreviations: Non-Motor Aspects of Experiences of Daily Living (nM-EDL), Motor Aspects of Experiences of Daily Living (M-EDL).

Outcome Measure	Change from baseline at week 12				Change from baseline at week 17			
	Active Mean	Placebo Mean	Therapeutic Gains	95% CI	Active Mean	Placebo Mean	Therapeutic Gains	95% CI
*Sample size*	*17*	*19*			*17*	*19*		
Movement Disorder Society Sponsored Unified Parkinson's Disease Rating Scale								
Part I: nM-EDL	−4.9	−2.1	−2.9	−5.7 to 0.0	−5.5	−0.2	−5.3*§	−7.9 to −2.7
Part II: M-EDL	−4.7	−0.5	−4.2*§	−6.4 to −2.0	−4.1	−0.2	−3.9*§	−6.6 to −1.2
Part III: Motor exam	−11.4	−3.9	−7.5*§	−13.2 to −1.8	−12.4	2.1	−14.5*§	−20.2 to −8.8
Postural Instability/gait difficulty +	−1.0	0.0	−0.5	−2.5 to 1.0	−1.7	−0.5	−1.1	−3.0 to 0.7
Non-Motor Symptom Scale								
Total Score	−36.1	−4.6	−31.5*	−49.5 to −13.5	−38.9	0.7	−39.6*	56.0 to −23.2
Domain 1: Cardiovascular +	0.0	0.0	1.5	0.0 to 3.5	0.0	−0.5	0.0	−1.0 to 3.0
Domain 2: Sleep/fatigue	−5.8	−4.4	−1.4	−6.0 to 3.2	−7.5	−2.6	−4.8*	−7.8 to −1.9
Domain 3: Mood/cognition	−10.8	3.4	−14.2*	−21.8 to −6.5	−10.3	4.3	−14.6*	−22.0 to −7.2
Domain 4: Perceptual +	0.0	0.0	0.0	−1.0 to 0.5	0.0	0.0	0.0	−1.0 to 0.5
Domain 5: Attention/memory	−6.0	−0.3	−5.7*	−10.3 to −1.1	−6.7	1.9	−8.7*	−13.3 to −4.0
Domain 6: Gastrointestinal tract	−5.2	−2.3	−2.9	−7.2 to 1.5	−5.5	−1.5	−4.0*	−7.4 to −0.6
Domain 7: Urinary	−6.6	−1.3	−5.3*	−10.4 to −0.2	−6.9	−2.8	−3.7	−9.1 to 1.7
Domain 8: Sexual function +	−1.8	0.0	−3.0*	−5.0 to 0.0	0.0	0.0	−3.0*	−6.0 to 0.0
Domain 9: Miscellaneous	−3.1	−0.6	−2.5	−5.5 to 0.6	−2.8	1.1	−3.9*	−7.0 to −0.8
Montreal Cognitive Assessment								
Total Score	2.5	0.4	2.0*	0.8 to 3.2	2.6	−0.2	2.7*	1.4 to 4.0
Hospital Anxiety and Depression Scale								
Anxiety Score	−1.5	−0.3	−1.2	−2.9 to 0.5	−2.5	−0.1	−2.5*	−4.2 to −0.8
Depression Score	−1.4	−0.2	−1.1	−2.5 to 0.2	−1.1	0.7	−1.9	−3.7 to −0.1
Epworth Sleepiness Scale								
Total Score	−2.0	0.2	−2.2	−4.9 to 0.5	−1.3	−0.8	−0.5	−3.1 to 2.1
Fatigue Severity Scale								
Total Score	−4.6	−1.3	−3.4	−10.2 to 3.4	−6.1	−0.2	−5.9	−12.0 to 0.2
Timed-up-and-go								
Time to complete +	−1.7	−0.2	−1.9	−5.6 to 0.8	−2.2	0.2	−1.5	−4.3 to 2.3
10 m walk								
Self-paced walk time (seconds) +	−0.8	0.0	−0.9*	−1.8 to −0.3	−0.8	0.5	−1.4*	−2.7 to −0.6
Fast-paced walk time (seconds)	0.2	0.2	0.0	−0.5 to 0.6	0.3	0.3	0.0	−0.6 to 0.5
2 minute walk								
Distance (meters)	0.2	−2.0	2.3	−10.3 to 14.8	5.9	−4.3	10.2	−1.2 to 21.6
Parkinson's Disease Questionnaire - 39								
Summary Index Score	−7.5	−4.4	−3.2	−7.9 to 1.6	−6.9	−2.3	−4.6	−9.3 to 0.0
Modified Schwab and England Activities of Daily Living Scale								
Total Score+	7.5	0.0	7.5*	0.0 to 15.0	7.5	0.0	7.5*	2.5 to 15.0
EQ-5D-5L								
Index Score	0.07	0.05	0.03	−0.05 to 0.10	0.02	−0.02	0.04	−0.06 to 0.13
SF-12 Health Survey								
Physical Composite Score	4.8	1.0	3.8	−0.9 to 8.4	2.9	0.7	2.2	−2.8 to 7.1
Mental Health Composite Score+	2.8	0.0	2.8	−2.1 to 7.6	2.9	−1.0	3.9	−1.3 to 9.2

**Table 4:** Adverse events. Abbreviations: Unrelated to device (UR); unlikely related to device (UlR); possibly related to device (PoR); probably related to device (PrR); related to device (R).

*Serious adverse events*		
Arm fracture	0	1 (UR)
Knee infection	1 (UR)	0
Fainting episode	1 (UlR)	0
*Other*		
Adverse drug reaction/side-effect, or complication of drug delivery	4 (3xUR, 1xUlR)	0
Arm injury	1 (UR)	0
Bowel dysfunction	1 (UlR)	0
Chest infection	0	1 (UR)
Confusion	1 (UR)	0
Depressive episode worsening	1 (UR)	0
Dizziness	3 (1xUR, 1xUlR, 1xPrR)	0
Ear discomfort/pain	2 (R)	0
Fall	1 (UR)	2 (1xUR, 1xUlR)
Freezing episode	1 (UR)	1 (UR)
Hypertension	0	1 (UR)
Hypotension	0	1 (UR)
Increased apathy	0	1 (UR)
Increased hallucinations	1 (UR)	0
Labyrinthitis exacerbation	0	1 (UR)
Migraine	1 (PoR)	1 (UR)
Motion sickness	1 (R)	0
Nasopharyngitis	1 (UR)	0
Nausea/vomiting	1 (UR)	0
Obtundation	1 (UR)	0
Post-elective surgery infection	1 (UR)	0

## 2 Experimental design, materials, and methods

PD patients were recruited from West and East Kent clinical neuroscience services and through the national PD charity *Parkinson's UK.* Eligibility criteria included the diagnosis of PD by the UK Parkinson's Disease Society Brain Bank Criteria along with reported limitations in activities of daily living. Additionally, patients needed to be receiving stable doses of dopaminergic drugs. Patients with previous exposure to neurostimulation or who were experiencing inner ear pathology were excluded.

### 2.1 Study design

Eligible subjects completed a 4-week baseline evaluation comprising assessments repeated in the first and fourth week and then were randomized (1:1) to active and placebo treatment groups. Subjects self-administered CVS at home (or if needed, with the help of a caregiver) for 8 weeks. Behavioral assessments were performed midway through the treatment period, at the end of treatment period and then 5 weeks after treatment cessation. All evaluations were conducted by the same blinded clinical researcher in subjects’ homes.

Ethical approval was obtained from the East Midlands NHS research ethics committee, and written informed consent was obtained from all subjects at study enrolment. The study was pre-registered at ClinicalTrials.gov as NCT02703844.

### 2.2 Stimulation protocol

For device treatments, subjects were instructed to lie on a 22°-elevated wedge pillow to orient the horizontal semi-circular canal vertically (thereby maximizing vestibular activation). Subjects self-administered CVS treatments twice daily using the solid-state TNM™ device previously described [2], [3], and were instructed to separate the two daily treatments by at least 1 hour. Active treatment involved the simultaneous delivery of a time-varying, warm, saw-tooth thermal (37 °C–42 °C) stimulus to one ear and a cold saw-tooth thermal (37 °C–17 °C) stimulus to the other ear for approximately 19 minutes (Fig. 2). Every 2 days, the warm and cold waveforms were switched between left and right ears to avoid the possible induction of a long-term, lateralized asymmetry in vestibular function. By slow warming and cooling of inner ear structures, it was possible to avoid vertigo and nausea that can result from chilled water irrigation used in the clinical testing of brainstem function. In the placebo testing condition, subjects underwent the same treatment choreography and experienced the same visual and auditory stimuli associated with running a treatment though no power was delivered to the heating and cooling elements. To maintain treatment blinding, treatment was discussed as brainstem modulation and no reference to the thermal stimulus was made. Subjects were told that they might or might not feel temperature changes in their ears (and that this would not be an indicator of active or placebo treatment; rather, temperature changes are naturally felt by some people and not by others). Subjects were told that they had a 50% likelihood of receiving either placebo or active treatment.

### 2.3 Validated outcome measures

Assessments were performed in the ON medication state (subjects reporting full medication effect against their Parkinsonism) and always timed to occur at the same time relative to the last dose of dopaminergic therapy. Outcome measures included the Movement Disorder Society-Unified Parkinson's Disease Rating Scale (MDS-UPDRS) [4], the Postural Instability and Gait Dysfunction score from the MDS-UPDRS [5], the Non-Motor Symptom Scale for PD [6], the Modified Schwab and England Activities of Daily Living Scale [7], the 2 minute walk test [8], the Timed-Up-and-Go test [9], the 10 m walk test [10], the Montreal Cognitive Assessment [11], the Hospital Anxiety and Depression Scale [12] the EQ-5D-5L [13], Epworth Sleepiness Scale [14], the Fatigue Severity Scale [15], the mental component summary score and physical component summary score of the Short-Form 12 (SF-12) [16] and the Parkinson's Disease Questionnaire-39 index summary score [17].

### 2.4 Statistical analyses

Analysis was conducted on the Intention-to-treat (ITT) dataset at the end of active treatment and at 5-week follow-up and included all randomized subjects (n = 46). Additional analysis was conducted on the modified intention-to-treat (mITT) data which included only those participants who completed the midway- but not end-of-treatment assessment. Results from the per protocol cohort are reported elsewhere [1]. Outcomes were analyzed using analysis of covariance (ANCOVA) to compare the change in the mean response (from the average of week 1 and 4 baseline scores) across treatment groups using an alpha of 0.05. The outcomes were adjusted for baseline symptom severity by including the baseline measure as a covariate. Outcomes with non-normal distributions were analyzed using Wilcoxon Rank Sum tests to compare the median change in response across treatment groups and the Hodges-Lehmann method was used to calculate median difference and confidence intervals. The Last Observation Carried Forward (LOCF) method was used to estimate missing values. The results are presented in Table 2, Table 3. Analysis was performed using SAS 9.4 (SAS Institute, Cary, NC, USA).

All randomized subjects were monitored for adverse events (AEs). Thirty four AEs were recorded, including 24 AEs in the active arm and 10 AEs in the placebo arm (Table 4). The likely cause of all AEs was determined by independent medical adjudication. All AEs that were deemed as potentially related to device use (n = 5) were non-serious and transient in nature (i.e. ceasing after termination of treatment).
